# A Cell Cycle Timer for Asymmetric Spindle Positioning

**DOI:** 10.1371/journal.pbio.1000088

**Published:** 2009-04-21

**Authors:** Erin K. McCarthy Campbell, Adam D Werts, Bob Goldstein

**Affiliations:** Department of Biology, University of North Carolina at Chapel Hill, Chapel Hill, North Carolina, United States of America; University of Cambridge, United Kingdom

## Abstract

The displacement of the mitotic spindle to one side of a cell is important for many cells to divide unequally. While recent progress has begun to unveil some of the molecular mechanisms of mitotic spindle displacement, far less is known about how spindle displacement is precisely timed. A conserved mitotic progression mechanism is known to time events in dividing cells, although this has never been linked to spindle displacement. This mechanism involves the anaphase-promoting complex (APC), its activator Cdc20/Fizzy, its degradation target cyclin, and cyclin-dependent kinase (CDK). Here we show that these components comprise a previously unrecognized timer for spindle displacement. In the Caenorhabditis elegans zygote, mitotic spindle displacement begins at a precise time, soon after chromosomes congress to the metaphase plate. We found that reducing the function of the proteasome, the APC, or Cdc20/Fizzy delayed spindle displacement. Conversely, inactivating CDK in prometaphase caused the spindle to displace early. The consequence of experimentally unlinking spindle displacement from this timing mechanism was the premature displacement of incompletely assembled components of the mitotic spindle. We conclude that in this system, asymmetric positioning of the mitotic spindle is normally delayed for a short time until the APC inactivates CDK, and that this delay ensures that the spindle does not begin to move until it is fully assembled. To our knowledge, this is the first demonstration that mitotic progression times spindle displacement in the asymmetric division of an animal cell. We speculate that this link between the cell cycle and asymmetric cell division might be evolutionarily conserved, because the mitotic spindle is displaced at a similar stage of mitosis during asymmetric cell divisions in diverse systems.

## Author Summary

Throughout animal development, and in stem cells, many cell divisions are asymmetric. The one-cell-stage C. elegans embryo divides asymmetrically, as a result of a displacement of the mitotic spindle to one side of the cell. As in other cell divisions, a mitotic progression machinery ensures that all chromosomes are associated with the metaphase plate before anaphase begins. This machinery involves the anaphase-promoting complex and its activator Cdc20/Fizzy, which target proteins for destruction by the proteasome; the cyclin that is targeted for degradation by the proteasome; and a cyclin-dependent kinase. We have asked whether the same machinery has a second function, delaying movement of the spindle to an asymmetric position until spindle assembly is complete. To address this question, we used genetic, reverse genetic, and pharmacological techniques to disrupt the function of elements of the mitotic progression machinery. We find that the mitotic progression machinery does indeed time spindle positioning, acting to delay spindle displacement until spindle assembly completes. This demonstrates a previously unrecognized link between the mitotic progression machinery and asymmetric spindle positioning in an animal cell.

## Abstract 3

The machinery that times entry into mitotic anaphase has the extra function during an asymmetric cell division of regulating when the mitotic spindle can shift to one side of a cell.

## Introduction

Asymmetric cell divisions often involve the asymmetric segregation of cell fate determinants as well as asymmetry in daughter cell size. Such asymmetry in daughter cell size typically results from the displacement of the mother cell's mitotic spindle to an asymmetric position within the cell before cytokinesis. Asymmetry in size of cells alone is likely to be important to partition determinants precisely [1], to allow large stem cells to divide repeatedly without becoming depleted of cytoplasm [2], and to permit meiosis in oocytes to produce small polar bodies and large eggs [3]. Asymmetric spindle positioning has been recognized for over a century [4,5], yet the mechanisms involved are only beginning to be elucidated [6].

**Figure 1:**
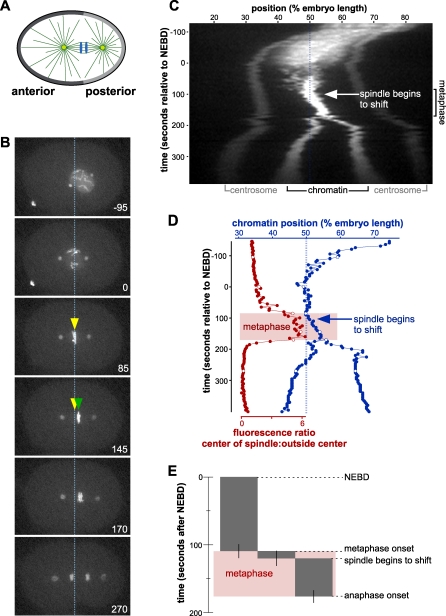
The Mitotic Spindle Begins to Shift Approximately 10 s after the Completion of Chromosome Congression (A) Diagram of the *C. elegans* zygote in anaphase, with chromatin represented in blue, microtubules in green, centrosomes in yellow, and anterior and posterior cortical PAR proteins in gray. (B) One-cell stage C. elegans embryo expressing histone H2B:GFP and gamma-tubulin:GFP, with time indicated in seconds before or after NEBD. Chromosomes complete congression to the metaphase plate at 50% embryo length (dotted blue line, yellow arrow) before the spindle becomes displaced posteriorly (green arrow). (C) Kymograph analysis of the embryo in (B), with the dotted blue line indicating 50% embryo length. The kymograph displays a time-series (*y*-axis) of a line from the anterior to posterior end of the embryo through the spindle (*x*-axis). (D) Quantitative analysis of the embryo in (B) and (C). Open circles at six timepoints correspond, from top to bottom, to the six timepoints in (B). Chromatin position is graphed in blue, and the degree of compactness of the chromatin is graphed in red, measured as a ratio of fluorescence intensities from the center of the spindle to directly outside this region, as detailed in Figure S1. Metaphase is indicated in pink. (E) Quantitative analysis of timing from 21 embryos imaged in multiple planes through the entire spindle and z-projected. Three intervals measured were from NEBD to metaphase onset as defined by completion of congression (left), from metaphase onset to the beginning of spindle displacement (middle), and from the beginning of spindle displacement to anaphase onset as defined by chromosome separation. The last two intervals define metaphase. Error bars represent the 95% confidence intervals for significance.

The mitotic spindle of the one-cell stage C. elegans embryo (Figure 1A) is moved to an asymmetric position by an inequality in microtubule pulling forces on the two sides of the spindle: The posterior cortex exerts greater net pulling forces on microtubules than the anterior cortex [7–9]. Several molecules that are critical for such pulling forces have been identified. Pulling forces depend on components of a protein complex that includes cortical G alpha protein, the mitotic spindle component LIN-5, and the posteriorly enriched activators of G protein signaling GPR-1/2. This complex recruits the microtubule motor dynein, which is also essential for strong pulling forces. There are a number of models for how these proteins, and analogous proteins in other model systems, result in stronger pulling forces on one side of a cell [6]. The transient enrichment of GPR-1/2 in the posterior cortex might increase the number of molecular links between dynein-associated microtubules and the cortex in the posterior. The G alpha, GPR-1/2, LIN-5 complex might also locally activate dynein motors, and/or promote handover of microtubules to dynein. Whether dynein functions here as a motor or only as a link to depolymerizing microtubules is not yet known. Other mechanisms may contribute to the inequality of pulling forces, including a PAR-dependent asymmetry in microtubule dynamics [10,11] and the local antagonism of G alpha-GPR-1/2 signaling by the DEP domain protein LET-99 [12]. Forces are temporally modulated during mitosis [9], although no temporal regulators have been reported to date.

We found previously that even before the spindle begins to move asymmetrically, two different kinds of forces hold the spindle in place. Pulling forces exist on the posterior side of the spindle, and these are balanced by a microtubule-based tether on the anterior side early in mitosis [9]. In the absence of the tether, the posteriorly directed pulling forces are sufficiently strong to move the spindle prematurely [9]. Pulling forces increase on both sides of the spindle near the time that spindle displacement begins [7,9]. We speculate, therefore, that a switch may exist to modulate forces and move the spindle at a precise time. Such a switch could be temporally regulated in a number of ways. Spindle displacement might be timed by links to the mitotic cell cycle. For example, the cell cycle machinery might affect the activity or localization of one or more of the cortical proteins discussed above, perhaps through the activity of one of the mitotic cyclin-cyclin-dependent kinase (CDK) complexes. Alternatively, mitotic progression and a progression of events at the cortex might run independently and in parallel. For instance, an intracellular signaling cascade at the cell cortex might affect the activity or localization of specific proteins that regulate spindle positioning. Such signaling could in principle result in active force generators being first activated beyond a threshold at a specific time after a developmental event such as fertilization, pronuclear meeting, or centration of the pronuclei.

We set out to test the hypothesis that mitotic progression regulates spindle displacement in an asymmetric cell division. C. elegans is an ideal system to explore this issue because it allows one to combine disruption of the mitotic progression machinery with microscopic imaging and precise quantification of chromosome and spindle dynamics. However, there has been a longstanding obstacle to studying this problem in C. elegans: Mitotic progression proteins are essential for successful completion of oocyte meiosis, such that disrupting these proteins' functions typically results in meiotic arrest before first mitosis [13–15]. This has precluded a number of straightforward tests of the hypothesis that mitotic progression regulates spindle displacement. Here, we report a set of genetic, reverse genetic, and pharmacological experiments, each designed to circumvent this obstacle for specific steps of a mitotic progression pathway. Our experiments tested whether mitotic progression pathway components are required for timely spindle displacement in embryos by disrupting the functions of individual components as well as multiple components for epistasis analysis. Cells with fluorescently tagged spindle components were used to analyze in detail spindle displacement and mitotic progression, and to analyze the consequences of misregulating the timing of spindle displacement. Our results demonstrate that anaphase-promoting complex (APC)-dependent negative regulation of CDK, long known to function as a mitotic progression timer, serves a second role as a timer for spindle displacement. We show that this timing mechanism ensures that spindle assembly is completed before spindle components begin to move away from the center of the cell.

## Results

### The Mitotic Spindle Moves to an Asymmetric Position at a Precise Time after the Completion of Chromosome Congression

There is disagreement about precisely when the mitotic spindle begins to shift to an asymmetric position in the C. elegans zygote. Some have stated that this begins at variable times before metaphase [16], during metaphase [17], or in late metaphase or anaphase [16,18–20]. Measurements have demonstrated that the spindle begins to shift well before anaphase begins [9,21].

We wished to determine precisely when the spindle begins to shift relative to the time when chromosome congression is completed, because we have observed in recordings that spindle displacement appears to closely follow completion of congression [9]. We therefore developed a method to quantitatively analyze the degree of chromosome congression and the position of the mitotic spindle simultaneously in individual embryos (Figures 1B–1D and S1). We first generated multiple-plane recordings through entire mitotic spindles in histone H2B:green fluorescent protein (GFP) and gamma-tubulin:GFP-expressing embryos [21] and tracked the movements of the chromosomes and both centrosomes. The degree of chromosome congression was quantified by measuring, at each timepoint, the ratio of histone-GFP fluorescence intensity in a small region at the center of the chromosome mass to histone-GFP fluorescence intensity just outside of this region. We calculated the center of the chromosome mass by a method that makes this ratio especially sensitive to single chromosomes unassociated with an otherwise complete metaphase plate (see Materials and Methods). This ratio peaks in metaphase (Figures 1D and S1). We found that spindle displacement toward the posterior began soon after chromosome congression was completed, early in metaphase (Figure 1B–1E and Video S1). This time is consistent with earlier measurements [9,21], and it additionally identifies the time when chromosome congression completes as the nearest recognizable mitotic event. The period from completion of chromosome congression to the beginning of anaphase chromosome separation lasted an average of 66.9 ± 8.8 (mean ± standard deviation) s. The spindle began to shift early during this period, and at a consistent time from embryo to embryo, starting 10.8 ± 11.3 s after we detected the completion of congression (Figure 1E).

### Proteasome Function Is Required for Timely Spindle Displacement

**Figure 2:**
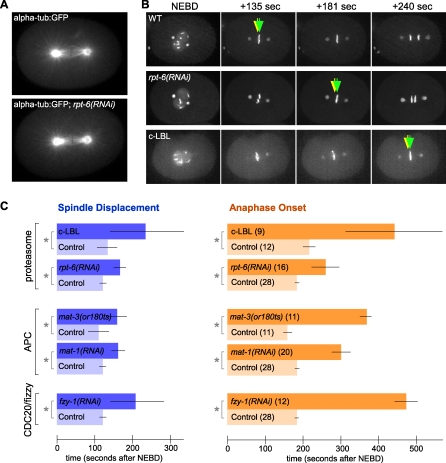
Proteasome Function, the APC, and Cdc20/Fizzy Are Required for Timely Spindle Displacement (A) Images from films of embryos expressing alpha-tubulin:GFP in control or *rpt-6(RNAi)* treated embryos, showing that RNAi targeting the proteasome component RPT-6 does not grossly disrupt spindle morphology. Using c-LβL to target proteasome activity produced similar results (unpublished data). (B) Images of embryos monitored for spindle displacement and anaphase onset, showing that treatments targeting the proteasome delay spindle displacement. Time is indicated in seconds after NEBD, and the chromatin position (green arrow) and 50% embryo length (yellow arrow) are indicated at the timepoint at which spindle displacement was first detectable for each case. (C) Quantitative analysis of the time between NEBD and either spindle displacement or anaphase onset, showing that disrupting proteasome function, the APC, or Cdc20/Fizzy results in a delay in spindle displacement. Components targeted are indicated at left, with specific treatments used to do so indicated on the data bars. Controls include the following, matched to each treatment: laser-permeabilized embryos in DMSO (for comparison to c-LβL), embryos in which the temperature was raised to 25 °C as in temperature-shift experiments (for comparison to *mat-3(or180)*), and embryos that were raised and imaged at 20 °C (for comparison to all other backgrounds in this figure). *mat-1(RNAi)* embryos were examined 3–6 h after injection. Treatments disrupting the APC or FZY-1 appear to specifically lengthen the times to anaphase onset and spindle displacement, and not to affect cell cycle timing more generally, as we found that they did not lengthen the interval from anaphase to cytokinesis (*n =* 11 embryos for *mat-1(RNAi)*, *n =* 7 embryos for *mat-3(or180)*, *n =* 7 embryos for *fzy-1(RNAi)*, *n =* 8 embryos for wild-type temperature-shifted control, *n =* 10 embryos for 25 °C wild-type control, *n =* 19 embryos for room-temperature wild-type control) nor shorten or lengthen the interval from pronuclear meeting to NEBD (*n =* 15 embryos for wild-type, *n =* 6 embryos for *mat-1(RNAi)*). The delay in anaphase onset after c-LβL treatment is less than that found previously [9]; here, we have used a lower concentration of c-LβL, which was sufficient to delay anaphase, and we have measured anaphase more precisely by observing histone H2B:GFP. Error bars represent the 95% confidence intervals for significance, and asterisks represent statistical significance at *p* < 0.05. Number of cases analyzed for each experiment is indicated in parentheses on the anaphase onset bars. For statistical values, see Methods.

The precise timing of spindle displacement relative to a mitotic event is consistent with our hypothesis that spindle displacement might be regulated by mitotic progression pathways. Mitotic progression depends in part on the degradation of specific proteins by the proteasome at the transition from metaphase to anaphase [22]. To determine directly if precise spindle displacement timing depends on proteasome activity, we disrupted the function of the proteasome by two methods that can circumvent a known requirement for the proteasome in meiosis [23]. First, to rapidly disrupt proteasome activity after meiosis was completed, we introduced a pharmacological inhibitor of the 20S proteasome's peptidase activity, *clasto*-lactacystin β-lactone (c-LβL) [24], to laser-permeabilized histone H2B:GFP and gamma-tubulin:GFP-expressing embryos. Second, we used *rpt-6* RNAi for specific targeting of a proteasome component in histone H2B:GFP and gamma-tubulin:GFP-expressing embryos. RPT-6 is a component of the 19S proteasome subunit, and its disruption has been shown to delay mitotic timing in the early embryo without disrupting meiosis [25]. We confirmed that disrupting proteasome activity by these methods did not prevent spindle formation or chromosome congression in alpha-tubulin:GFP-expressing embryos and in histone H2B:GFP and gamma-tubulin:GFP-expressing embryos (Figure 2A and 2B). As expected, both *rpt-6* RNAi and c-LβL caused delays in anaphase onset (Figure 2B and 2C). In support of our hypothesis, we found that both of these treatments also delayed spindle displacement toward the posterior (Figure 2B and 2C; Video S2), suggesting that proteasome function is required for timely spindle displacement. This effect was not reported in our earlier experiments using c-LβL [9], but our previous observations were made by Nomarski imaging of spindles alone, and the timepoints examined earlier would not have revealed a short delay. Because proteasome disruption is likely to have a wide spectrum of direct and indirect effects in cells, we next considered whether components of the mitotic progression machinery with more specific roles regulate the timing of spindle displacement.

### The APC Is Required for Timely Spindle Displacement

The proteasome has a large number of targets, a subset of which are tagged for degradation by the APC, a multi-subunit E3 ubiquitin ligase that regulates the timing of anaphase through specific targets [22]. To test whether the APC temporally regulates spindle displacement, we disrupted the functions of C. elegans homologs of two key components of the APC. Because the APC is required for progression through meiosis in C. elegans, we used methods that can allow meiotic progression and then disrupt mitosis. First, we used a fast-acting temperature-sensitive allele of *mat-3*, the C. elegans homolog of APC8/CDC23 [14]. We crossed a histone H2B:GFP transgene into *mat-3(or180ts)* and shifted embryos to the restrictive temperature only after meiosis, just prior to mitosis. This produced a delay in anaphase onset, as expected. We found that this also delayed spindle displacement (Figures 2C and S2). Second, we used carefully timed dsRNA injections into histone H2B:GFP and gamma-tubulin:GFP hermaphrodites to attempt partial depletion of MAT-1, the C. elegans homolog of APC3/CDC27. We found a time window after injection of *mat-1* dsRNA when embryos progressed through meiosis successfully, as judged by the presence of only two pronuclei in the embryo before mitosis began, and anaphase onset was delayed in mitosis. We found that spindle displacement was delayed as well (Figures 2C and S2). The length of the intervals between pronuclear meeting and nuclear envelope breakdown (NEBD) and between anaphase and cytokinesis were unaffected, suggesting that the effect we see is not an indirect effect on cell cycle timing more generally (Figure 2, legend). On the basis of these results, and on results below that show suppression of the spindle displacement delay by prematurely inactivating a known APC target, we conclude that the APC is required for timely spindle displacement.

### CDK Inactivation as a Timer for Spindle Displacement

**Figure 3:**
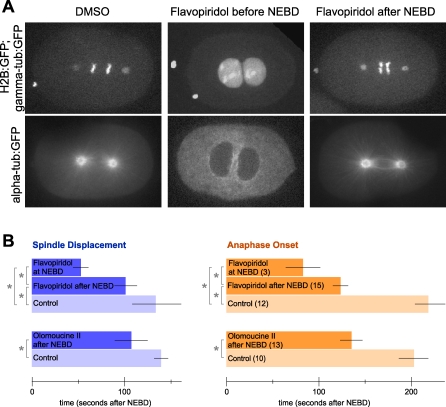
Premature CDK Inactivation Results in Premature Spindle Displacement (A) Embryos expressing histone H2B:GFP and gamma-tubulin:GFP, or expressing alpha-tubulin:GFP, were laser-permeabilized. Embryos permeabilized in DMSO proceeded through mitosis similar to wild-type, and had normal spindle morphology. When embryos were treated with flavopiridol prior to mitosis, they did not undergo NEBD (11/12 H2B:GFP- and gamma-tubulin:GFP-labeled embryos), nor did they maintain centrosome-nucleated microtubules (8/9 alpha-tubulin:GFP-labeled embryos). Embryos treated with flavopiridol later in mitosis proceeded through mitosis with premature anaphase (below), and with normal spindle morphology (8/8 alpha-tubulin:GFP-labeled embryos). (B) Flavopiridol treatment at the start of or after NEBD, or olomoucine II treatment after NEBD, caused spindle displacement and anaphase onset to occur earlier than in wild-type. Controls are laser-permeabilized embryos in DMSO. Flavopiridol-treated embryos were examined to determine if the treatment specifically shortened the times to spindle displacement and anaphase onset, and not speed up cell cycle timing more generally: Treatment did not shorten the interval from anaphase to cytokinesis (*n =* 19 embryos for wild-type control, *n =* 4 embryos for flavopiridol). Error bars represent the 95% confidence intervals for significance, and asterisks represent statistical significance at *p* < 0.05. Number of cases analyzed for each experiment is indicated in parentheses on the anaphase onset bars. For statistical values, see Methods.

If mitotic progression components serve as a bona fide timer for the onset of spindle displacement, rather than just being required for the efficient execution of spindle displacement, then the converse effect on timing should be possible: Premature inactivation of a critical APC target should result in premature spindle displacement. Cyclin B is an important target of the APC in mitotic progression, and degradation of cyclin B inactivates CDK [26]. Although it has not yet been possible to visualize directly the inactivation of CDK in C. elegans embryonic mitoses, these events are essentially universal in animal cell mitoses, and degradation of GFP-tagged cyclin B has been observed in C. elegans during meiosis [26–28]. We considered a number of methods to alter the timing of CDK inactivation in mitosis. Loss of maternal CDK in C. elegans mutants or by RNAi results in meiotic defects before first mitosis [29], and introducing a nondegradeable form of cyclin B would be expected to do the same. To inhibit CDK activity at specific times, we laser-permeabilized embryos to a highly specific pharmacological inactivator of CDK, the anticancer drug flavopiridol [30,31]. First, we performed two sets of functional tests of flavopiridol's efficacy on C. elegans embryos. (1) We determined whether flavopiridol treatment of alpha-tubulin:GFP embryos before NEBD could result in phenotypes consistent with CDK inactivation. We applied the drug to one-cell stage embryos prior to NEBD by laser-permeabilization of embryos at this stage. Because a cyclin-CDK complex promotes entry into mitosis, inhibition of CDK activity at this early stage should block mitotic entry [26]. Consistent with this, we found that NEBD failed to occur, and most microtubules were found unassociated with centrosomes (Figure 3A). (2) We next treated embryos with flavopiridol after NEBD. Flavopiridol treatment at this time had no apparent effect on microtubules or the mitotic spindle (Figure 3A), suggesting as expected that the dramatic effect of earlier treatment on microtubules was an indirect effect of blocking mitotic entry. Although the time window from NEBD until the normal time of anaphase is short, about 3 min, we found that flavopiridol treatment after NEBD succeeded in causing premature anaphase onset (Figure 3B). On the basis of the effects of flavopiridol on mitotic entry and anaphase timing, we conclude that flavopiridol is likely to be an effective inhibitor of CDK activity in C. elegans embryos, as it is in diverse animal and protozoan systems [30–32].

Flavopiridol treatment after NEBD resulted in anaphase bridges in some embryos, although most embryos succeeded in separating chromosomes completely (10/14 cases). The ability of chromosomes to separate early in many cases suggests that CDK inactivation is likely to promote chromosome separation in C. elegans by regulating separase activity, as in certain other systems [33]. Importantly, we found that flavopiridol treatment also caused the spindle to shift earlier than it would normally do so (Figure 3B and Video S3). We found that slightly earlier flavopiridol treatment, just as NEBD began, produced similar but more dramatic results, including earlier anaphase onset, more frequent anaphase bridges (8/11 cases), and earlier spindle displacement—about 60–90 s earlier (Figure 3). The length of the interval from NEBD to spindle displacement was less than half as long in these embryos as in untreated embryos (Figure 3A), yet the length of the interval between anaphase and cytokinesis was not shortened, suggesting that the effect we see is not an indirect effect of speeding up development or cell cycle timing more generally (Figure 3, legend). We treated embryos with a different CDK inhibitor, the purine analog olomoucine II [34], and found that this too resulted in earlier spindle displacement and earlier anaphase (Figure 3B). Since we found that targeting the APC can delay spindle displacement, and targeting CDK can produce the converse effect, we conclude that APC-dependent regulation of CDK activity is likely to serve as a bona fide timer for spindle displacement. To further test this hypothesis, we next asked whether a known substrate determinant used by the APC to target cyclin is involved, and we determined whether the delay caused by inactivating the APC depends on active CDK.

### FZY-1/Cdc20 Regulates Spindle Displacement Upstream of the APC

A key activator of the APC in mitosis is the tryptophan-aspartic acid repeat protein Cdc20/Fizzy. Cdc20/Fizzy is inhibited by a set of kinetochore proteins, the spindle checkpoint proteins, until all kinetochores are attached to the spindle. Upon checkpoint inactivation, Cdc20/Fizzy is able to bind to and activate the APC, serving as a substrate determinant for the APC to target both cyclin B and securin for degradation [35]. We were interested in determining whether the same substrate determinant is relevant to the APC's function in timing spindle displacement.

We considered several approaches for testing the roles of Cdc20/Fizzy and its regulators, the spindle checkpoint proteins, in timely spindle displacement. Experiments that compromise spindle assembly have been valuable for studying regulation of anaphase timing in various systems [36], but cannot be used to study spindle displacement because experimentally compromised spindles are known to move aberrantly under early-acting cortical forces [9]. We therefore considered targeting spindle checkpoint genes in embryos with intact spindles. The spindle checkpoint was first defined by the yeast (mitotic arrest deficient) *MAD* genes [37] and (budding uninhibited by benomyl) *BUB* genes [38]. Both sets of genes were named for their ability to bypass mitotic arrest caused by the microtubule-destabilizing drug benomyl, and neither of the screens that identified these genes required them to have essential functions in normal yeast. Indeed, null alleles of some *MAD* and *BUB* genes grow at wild-type rates [37–39]. This has indicated that some of the core functions performed by the checkpoint proteins are nonessential functions, required only when spindle assembly is compromised. For this reason, experiments targeting checkpoint proteins alone, in the absence of spindle damage, sometimes do not reveal the roles of checkpoint proteins in regulating anaphase timing.

We found that loss of MDF-2/Mad2 or MDF-3/Mad3 can delay spindle displacement (Figure S3A). However, it appears unlikely that the delays we observed can be explained solely by these proteins' well-known roles upstream of the APC and CDK, since by flavopiridol treatment of an *mdf-3* mutant, much of the delay appears to be CDK-independent (Figure S3B), and because anaphase timing was not similarly affected in the absence of spindle damage (Figure S3A) [40]. We predicted, therefore, that the effect we observed on the timing of spindle displacement might reflect a dominant and potentially nonphysiological result of releasing active FZY-1/Cdc20 at a time when it would never normally be active. Indeed, we found that a gain-of-function allele of *fzy-1* that behaves genetically as a constitutively active allele [41] produced the same results, delaying spindle displacement in a manner that appeared partially CDK-independent by flavopiridol treatment of the gain-of-function *fzy-1* mutant (Figure S3). There is precedent for Cdc20/Fizzy functioning independently of the APC in other systems, although the mechanism by which it does so is not understood by us or by others [42].

Given these results, we decided to pursue the function of FZY-1 by disrupting its function, rather than by gain of function or by disrupting its negative regulators. Because the C. elegans Cdc20/Fizzy protein FZY-1 is required for meiotic progression before mitosis, we attempted to bypass its requirement in meiosis by partial depletion of FZY-1 using timed dsRNA injections. At 10 h postinjection, we found that *fzy-1* dsRNA succeeded in consistently and significantly delaying anaphase onset at first mitosis without causing meiotic arrest or failure (12/12 embryos). This treatment also significantly delayed spindle displacement (Figures 2C and S2). Together with results above, this suggests that FZY-1, the APC, and proteasome activity are required for timely spindle displacement. We next considered whether these components function in timing spindle displacement by the same pathway they rely on in timing anaphase.

### The APC and the Proteasome Time Spindle Displacement Primarily through Negative Regulation of CDK

**Figure 4:**
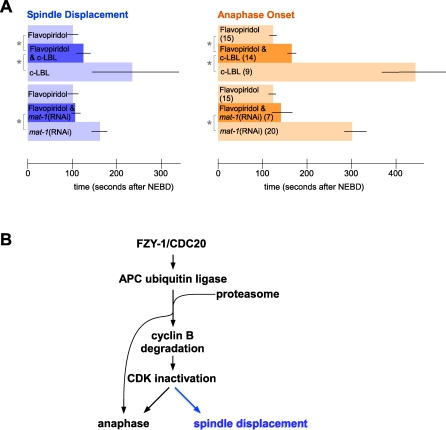
The Proteasome and APC Function in Spindle Displacement Primarily Through CDK Inactivation (A) Flavopiridol treatment rescued most of the delay of anaphase onset and spindle displacement induced by the proteasome inhibitor c-LβL. Flavopiridol treatment completely rescued the delays induced by *mat-1* RNAi, for both anaphase onset and spindle displacement. Error bars represent the 95% confidence interval for significance, and asterisks represent statistical significance at *p* < 0.05. Number of cases analyzed for each experiment is indicated in parentheses on the anaphase onset bars. For statistical values, see Methods. (B) A working model for regulation of spindle displacement (blue) by the Cdc20/Fizzy, APC, cyclin-CDK pathway (black). We predict that a known or unknown molecule that functions in spindle displacement may be a direct or indirect target of CDK activity.

Our data suggest that negative regulation of CDK by the APC times spindle displacement. However, the APC targets other proteins for degradation in addition to cyclin B [26]. To determine whether the proteasome and the APC time spindle displacement primarily through negative regulation of CDK, we determined whether flavopiridol treatment could rescue the delay caused by disrupting the proteasome and the APC. First, we used c-LβL to disrupt proteasome function in one-cell stage embryos as before, and we then added flavopiridol after NEBD. Flavopiridol rescued most of the c-LβL-induced anaphase delay, and we found that flavopiridol also rescued most of the spindle displacement delay (Figure 4A). These results suggest that although c-LβL might have off-target effects, and although proteasome disruption should affect many processes, the effects of this drug on delaying anaphase and spindle displacement are primarily CDK-dependent. Second, we disrupted *mat-1* by RNAi as before and treated these embryos with flavopiridol. We found that the delays in both anaphase onset and spindle displacement were completely rescued (Figure 4A). We conclude that the proteasome and the APC time spindle displacement in the one-cell C. elegans embryo primarily through their roles in inactivating CDK. Taken together, our results suggest that the timing of mitotic spindle displacement in this system is regulated by the well-studied pathway involving the APC and proteasome activity acting upstream of, and inhibiting, CDK activity (Figure 4B). Since each experiment that affected the timing of anaphase similarly affected the timing of spindle displacement, but spindle displacement preceded anaphase in each such experiment, we conclude that CDK inactivation has a more rapid effect on spindle displacement than on anaphase. An alternative explanation, however, is that anaphase timing may depend on timely spindle displacement in this system. We test this below.

### Anaphase Onset Timing Does Not Depend on Spindle Displacement in the *C. elegans* Zygote

**Figure 5:**
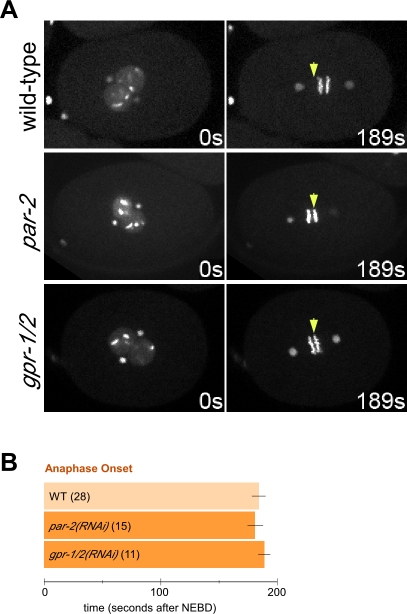
The Time of Anaphase Onset Does Not Depend on Spindle Displacement (A) Time of NEBD (0 s) and soon after anaphase onset (189 s) in wild-type, *par-2(RNAi)*, and *gpr-1/2(RNAi)* embryos. Yellow arrows mark 50% embryo length. *par-2(RNAi)* and *gpr-1/2(RNAi)* spindles did not become displaced toward the posterior. Anaphase began at roughly the same time as in wild-type. (B) Quantitative analysis of time from NEBD to anaphase onset in wild-type and for embryos in which spindle displacement did not occur, in *par-2(RNAi)* and *gpr-1/2(RNAi)*. Anaphase onset timing in *par-2(RNAi)* and *gpr-1/2(RNAi)* embryos were statistically indistinguishable from anaphase onset timing in wild-type embryos (*p =* 0.48 and *p =*0.33, respectively). All embryos express H2B:GFP and gamma-tubulin:GFP, and only embryos that divided equally after RNAi were analyzed. Number of cases analyzed for each experiment is indicated in parentheses.

In certain systems, spindle position is monitored by the cell cycle machinery, with aberrant spindle positioning causing a delay in the cell cycle [43]. For example, spindle misorientation in rat epithelial cells causes a short delay in anaphase [44]. Given this and our results above, we hypothesized that precise anaphase timing might depend on spindle displacement in the zygote. Although spindle displacement occurs at a precise time in the C. elegans zygote, to our knowledge, there has been no direct test of the hypothesis that anaphase may be delayed in the absence of spindle displacement. We tested this by genetically preventing spindle displacement and monitoring anaphase timing. Spindle displacement was prevented by disrupting the function of the G protein regulators GPR-1/2 by RNAi in histone H2B:GFP and gamma-tubulin:GFP-expressing embryos. We similarly disrupted the function of PAR-2, a ring finger protein that functions in polarity establishment and maintenance before spindle displacement [45]. The effectiveness of RNAi was confirmed by examining embryos for a failure in spindle displacement. We found that anaphase occurred in each background at a time that was statistically indistinguishable from that in normal embryos (Figure 5 and Video S4). We conclude that spindle displacement is not required for accurate anaphase timing in *C. elegans*. The results suggest instead that CDK activity affects spindle displacement and anaphase timing independently (Figure 4B).

### The Consequence of Misregulating the Timing of Spindle Displacement

**Figure 6:**
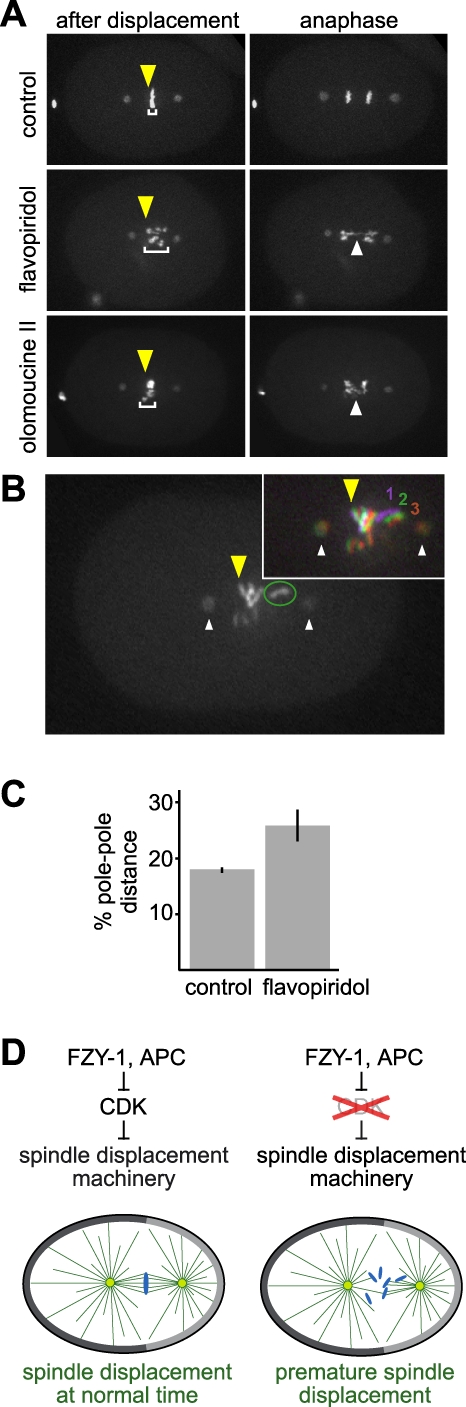
Flavopiridol Treatment Results in the Displacement of Incompletely Assembled Mitotic Spindles (A) Images of untreated, flavopiridol-treated, and olomoucine II-treated embryos after spindle displacement and in anaphase. The chromosomes have a wider distribution (brackets) along the anterior-posterior axis at the time of spindle displacement after flavopiridol or olomoucine II treatment. Yellow arrows indicate 50% embryo length. White arrows point to chromosome bridges in anaphase, likely to be a result of premature anaphase. (B) Movement of a chromosome toward a spindle pole after spindle displacement has begun in a flavopiridol-treated embryo. One chromosome (circled) is tracked in three color-coded timepoints (numbered 1, 2, 3) in the inset. Timepoints shown are 3 s apart. Yellow arrows indicate 50% embryo length, and white arrows indicate centrosomes. (C) Measurements of the degree of chromosome congression at the time of spindle displacement. After treating with flavopiridol after NEBD, chromosomes were not aligned on the metaphase plate as tightly as in WT. For statistical values and *n*-values, see Methods. (D) Model for the role of CDK regulation of spindle displacement: We speculate that a component of the spindle displacement machinery may be a direct or indirect target of CDK activity and that this produces the temporary delay in spindle displacement. CDK regulation of spindle displacement timing permits spindle assembly to complete before movement begins. Left, normal temporal regulation results in spindle displacement after congression completes; right, premature spindle displacement resulting from experimentally inactivating CDK early results in spindle displacement before congression completes. Colors are as in Figure 1A.

To determine the biological function of the mechanism that times spindle displacement, we examined the consequence of unlinking spindle displacement from normal temporal regulation of CDK activity by the APC. Spindle displacement normally begins just seconds after chromosome congression. Therefore, premature spindle displacement in flavopiridol- or olomoucine II-treated embryos should produce a potentially important developmental consequence—shifting the spindle before all chromosomes become properly attached and aligned at the metaphase plate. Indeed, we found that upon treatment with either drug, as the spindle began to shift, spindles were only partially assembled. In some cases, one or more chromosomes were not yet associated with the metaphase plate as premature spindle displacement occurred (Figure 6A), and we found that even after spindle displacement began, chromosome movement could still be directed away from the metaphase plate, toward one of the spindle poles (Figure 6B). These results suggest that not all chromosomes have received a full complement of force-producing microtubule attachments from both spindle poles by the time of premature spindle displacement. As expected given these results, we found that flavopiridol treatment after NEBD resulted in chromosomes being spread on average over a significantly wider area than normal at the onset of premature spindle displacement (Figure 6C and Videos S5 and S6). In many cases, chromosomes never completed congression (6/8 cases upon treating at NEBD, 0/21 in untreated embryos, *p* < 0.05). These results suggest that normal temporal regulation of spindle displacement serves to ensure that spindles are fully assembled, with chromosomes aligned at the metaphase plate and with a full complement of force-producing microtubule attachments from each spindle pole, before spindle displacement begins.

## Discussion

We have shown here that the timing of spindle displacement is precisely regulated in the C. elegans zygote. We combined quantitative measurements of events in mitosis with genetic, reverse genetic, and pharmacological techniques to test whether a link exists between the roles of the APC and CDK in mitotic progression and mitotic spindle displacement. Our results demonstrate that negative regulation of CDK activity by the proteasome, FZY-1, and the APC serves as a previously unrecognized timer for mitotic spindle displacement. This regulation of spindle displacement by CDK activity results in an approximately 60–90-s delay in spindle displacement—a delay that may be important, because it allows spindle assembly to complete before cortical forces pull the spindle away from the center of the cell (Figure 6D, model).

How might CDK inactivation impinge on the mechanism of spindle displacement? The mechanism by which mitotic spindles become positioned asymmetrically is a topic of intense interest. In a number of animal systems, proteins involved in spindle displacement have been identified, including a set that acts downstream of general polarity regulators such as the PAR proteins [6]. Myristoylated, membrane-anchored G alpha proteins have been found to associate with G protein regulators, which serve as key links to mitotic spindle proteins—to the spindle protein NuMA in mammals, a NuMA-related protein Mud in flies, and another spindle-associated protein, LIN-5, in C. elegans [12,46–53]. Two key findings—that cortical G protein regulators can be asymmetrically positioned in worms and flies, and that these and LIN-5 associate with dynein complex components—have prompted a number of models for how an asymmetric distribution of G protein regulators might position the spindle in worms and flies [6,20,54]. How these components result in asymmetric forces on the spindle beginning in early metaphase remains a fascinating and incompletely understood issue. Given the results we report here, it will be important to determine if any of the proteins that are required for spindle displacement are temporally regulated by CDK-dependent phosphorylation. GPR-1/2, LIN-5, and LET-99 have distributions that are dynamic through mitosis, and these dynamic distributions might be temporally regulated directly or indirectly by CDK activity, although no such link is yet known [12,47,49,51,55]. Interestingly, work in *Xenopus* extracts has shown that NuMA is dephosphorylated and released from spindle poles through CDK inactivation [56].

It is also possible that additional players in asymmetric division have yet to be identified, and that one or more of these could be relevant CDK targets. A microtubule motor that functions in the C. elegans zygote, the MKLP kinesin-like protein ZEN-4, is temporally regulated by CDK phosphorylation, although ZEN-4 is not known to play an essential or redundant role in spindle displacement [57]. Separase, an APC- and CDK-regulated protease involved in separating chromosomes, has multiple targets, including some not involved in chromosome separation [58], for example in disengaging duplicated centrioles and in C. elegans cortical granule exocytosis [59,60]. Although there is no current evidence that separase functions in spindle displacement, it is at least conceivable that separase targets might include one or more proteins involved in positioning the mitotic spindle. In yeast, CDK and cyclin B proteins directly or indirectly regulate asymmetries in the distribution of dynein and the cytoskeletal-associated protein Kar9 [61–63]. C. elegans does not have a Kar9 homolog, and dynein distribution appears symmetric in the *C. elegans* zygote [64], although dynein is present on so many structures in the *C. elegans* zygote that if a subtle but biologically relevant asymmetry in cortical dynein levels exists, it might be difficult to detect.

It is not yet clear what acts upstream of CDC20/Fizzy to time spindle displacement. Spindle-assembly checkpoint components are reasonable candidates, given that they regulate CDC20/Fizzy in various systems, and given that we have implicated *fzy-1* and downstream proteins in spindle displacement at a time just after the spindle-assembly checkpoint is normally satisfied [36]. Early-stage animal embryos have long been said to lack many cell cycle checkpoints [65], but multiple exceptions have been found in which checkpoints can cause delays in early embryonic cell cycles, delays that are typically much shorter than in nonembryonic cell cycles [40,66,67]. Spindle-assembly checkpoint proteins have been shown to function in the early C. elegans embryo by experiments in which spindle integrity was compromised in wild-type and mutant backgrounds [40]. As discussed, it can be difficult to discern the complex roles of spindle-assembly checkpoint proteins in the absence of damage to the spindle itself. We have made several attempts to cause more subtle insults to spindle integrity. Microtubule attachments to kinetochores appear to be important structural components of the spindle in C. elegans, as the spindle is pulled apart early in kinetochore-null mutants [21]. We tried to create situations where only some but not all chromosomes are unattached, by several methods: by partial RNAi depletion of kinetochore components, by introducing extra chromatin fragments by DNA injection, or by introducing only one set of kinetochore-deficient chromosomes through the sperm (EKMC and BG, unpublished data). As might have been expected, none of these methods caused a delay in anaphase without also compromising spindle integrity. In the absence of positive evidence that FZY-1 is responding to the spindle-assembly checkpoint, the possibility that FZY-1 is regulated in another way is a viable alternative. Other possible regulators of FZY-1 include homologs of yeast Emi1, the mammalian centrosomal protein RASSF1A, and the mammalian sperm protein speriolin, all of which can regulate CDC20/Fzy in other systems [68–70]. Despite this limitation in identifying FZY-1 regulators in spindle displacement, the consequence of misregulating spindle displacement timing is clear—spindles can move to an asymmetric position before spindle assembly is completed (Figure 6).

Given our finding that unlinking spindle displacement from the timing mechanism we describe results in displacement of partially formed spindles, it seems plausible that a link between mitotic progression and spindle displacement evolved to ensure that spindle assembly is complete before the spindle shifts. Delaying spindle displacement until spindle assembly completes may have been evolutionarily advantageous because it might help prevent a low frequency of chromosome loss during spindle displacement. Chromosome loss is an important contributor to tumorigenesis [36]. Mutations in several APC components have been implicated in human cancers, and failures of multiple functions of the APC have been suggested as the basis for these mutations in carcinogenesis [71]. We speculate that asymmetric divisions, for example in stem cells, may have an added burden to prevent chromosome loss as the spindle moves to one side of a cell, and that such movements as well as premature anaphase may contribute to chromosome loss in some cancers.

Our finding that the APC and CDK activity time spindle displacement identifies a new link between cell biology and development. Chromosomes first adopt an asymmetric position during M-phase of asymmetric divisions in diverse systems, including *Drosophila*, *C. elegans*, annelids, and mammals. Examples include neuroblast divisions in *Drosophila* [72] and leech [73], sensory organ precursor divisions in *Drosophila* [74], early embryonic cell divisions in leech [75] and *Tubifex* [76,77], and meiosis in mouse oocytes [78]. It is perhaps striking that in all of these, chromosomes are first positioned asymmetrically soon after chromosome congression, during metaphase or anaphase [72–78]. It will be interesting to learn whether mitotic progression is an evolutionarily conserved temporal regulator of spindle positioning in asymmetric cell divisions.

## Materials and Methods

### C. elegans strains.

Published strains used in this study include the following: TH32 (*unc-119(ed3)* III; ruIs32[*unc-119*(+) *pie-1*::GFP::histoneH2B]; ddIs6 [*unc-119*(+) *pie-1*::GFP::TBG-1]), AZ212 (*unc-119(ed3)* III; ruIs32[*unc-119*(+) *pie-1*::GFP::histoneH2B]; ddIs6) and OD3 (ltIs24[pAZ132; *pie-1*::GFP::TBA-2 + *unc-119*(+)], a gift from Paul Maddox), cultured at 20 °C. For imaging of the checkpoint and APC alleles, strains of *mdf-2(av16)*, *mdf-3(av20)*, *fzy-1(av15)*, and *mat-3(or180)* (gifts from Andy Golden) were crossed into TH32 or AZ212. Checkpoint alleles were cultured at 24 °C, and *mat-3(or180)* was cultured at 15 °C and moved to 25 °C 1 min prior to experiments and recorded at 25 °C.

### RNA interference.

*mat-1* and *fzy-1* functions were disrupted by injecting dsRNA as described previously [79], and imaging embryos at multiple time points after injection to identify a time when embryos reached first mitosis without meiotic defects, but had a delay in anaphase timing. *rpt-6* function was disrupted by feeding bacteria expressing dsRNA, and *par-2* was disrupted by injecting dsRNA as described previously [79,80]. *gpr-1/2* were targeted by dsRNA to *gpr-2* alone. As the DNA coding sequences for GPR-1 and GPR-2 are nearly identical [47,49,53], this should disrupt the functions of both genes.

### Drug treatments*.*

To permeabilize embryos for drug treatment, embryos were mounted in a drug on poly-L-lysine-coated and washed coverslips, with clay feet used as spacers, coated in small pieces of charcoal, and sealed with valap (equal parts petroleum jelly, lanolin, and paraffin). Charcoal pieces attached to the eggshell were targeted with a 2-mW pulsed laser (model VSL-337; Laser Science Inc.) containing Coumarin 440 dye in a lasing chamber (Photonic Instruments), to produce small holes in the eggshell. Embryos were treated with the following drugs: 20 μM c-LβL (Calbiochem), 200 μM flavopiridol (NCI) for experiments prior to the entry into mitosis, and 400 μM flavopiridol or 2 mM olomoucine II (Sigma) for experiments during mitosis. As each drug was stored in DMSO, controls were carried out in egg buffer and the appropriate amount of DMSO for each drug. Such high concentrations of drugs were used because laser permeabilization of C. elegans embryos generally results in only a small hole in the eggshell. To treat embryos with flavopiridol or olomoucine II during mitosis, slides were mounted in egg buffer and sealed on only two sides. At a specific time, the drug was added to an unsealed side, while egg buffer was wicked from the other side. For the experiment in which flavopiridol was used to rescue the effects of c-LβL, embryos were permeabilized in c-LβL. During mitosis, a combination of both drugs was washed into the chamber to avoid washing out the c-LβL when washing in the flavopiridol.

### Imaging and analysis.

Embryos (other than drug-treated embryos) were mounted as described previously [9]. Time-lapse images were acquired using a CSU10 Yokogawa spinning-disk confocal system (McBain) mounted on an inverted microscope (Eclipse TE2000; Nikon). The embryos were illuminated at 488 nm with a 50-mW air-cooled Argon laser (Laser Physics). Digital images were acquired by a 16-bit cooled CCD camera (Orca ER; Hamamatsu), and the acquisition system was controlled by MetaMorph software (Universal Imaging Corporation). For quantifying the duration of events in mitosis, images were acquired with 650-ms exposure at 3-s intervals. Images for multiplane z-series were acquired at 5-s intervals with 400-ms exposure time, in five steps of 1.25 μm each. All images were acquired using 100× Plan Apochromat VC NA1.4 or 60× Plan Apochromat NA1.4 objectives, and 2 × 2 binning in the camera. Images were analyzed using MetaMorph software and Microsoft Excel, and processed in Photoshop (Adobe Systems).

To quantitatively assess the degree of chromosome congression as spindle displacement began, we measured fluorescence intensity, using MetaMorph, from histone H2B:GFP; gamma-tubulin:GFP embryos along the length of a rectangular box running from the anterior to the posterior end of the embryo through the width of the chromatin in the plane of view, and through a projection of the entire spindle in all of the z-planes recorded. Fluorescence intensities were exported to Microsoft Excel, and further analysis was carried out in Microsoft Excel. Chromatin position was identified as the peak position of a 13-pixel-wide running average of fluorescence intensity values (or 5-pixel wide for one timepoint at anaphase to better resolve anaphase separation of chromatin), and two peaks were identified similarly after anaphase. The pixel size used was 0.14 μm. The degree of compactness of the chromatin before, during, and after metaphase is reported as the fluorescence signal ratio at the center of the spindle versus that just outside (Figure S1), obtained by collecting average pixel value along a 13-pixel-wide region at the center of the chromatin position (defined here as the peak value a 31-pixel-wide running average) and average pixel value for two 16-pixel-wide regions on either side of the center region, subtracting from each the background level of fluorescence, defined as the minimum pixel intensity value of a 158-pixel-wide region in the center of the embryo. These region widths were selected to ensure that the values produced were sensitive to individual chromosomes out of the metaphase plates observed in several recordings. To quantify the progress of chromosome congression as in Figure 6, the width of the area in which chromosomes reside in the spindle was calculated as a percentage of the spindle pole-pole distance.

To analyze the timing of NEBD in embryos, we measured the fluorescence intensity of the histone H2B:GFP signal. NEBD was defined as the time when the fluorescence intensity (defined as pixel intensity within a 20 × 20 pixel square positioned in an area of the nucleus free of a chromosome minus a 20 × 20 pixel square of background within the embryo) dropped to 50% the initial measurement. Chromosome congression (Figure 1) was defined as the time when the chromosome mass resided within 15% of the distance between spindle poles. The beginning of spindle displacement was defined as the time when the chromosomes moved to 52% embryo length and did not return past this mark. Anaphase onset was defined as the time when the single chromosome mass first became resolvable as two masses.

Kymographs (Figure 1) were created using Metamorph software, using an 80-pixel-tall line that spanned the embryo's length, calculating average intensities at each time frame.

### Statistics.

We used two-tailed *t*-test *p*-values to determine significance in all experiments. For experiments represented in Figure 2, the *p*-values and *n*-values were the following: For treatments in which the proteasome was disrupted, anaphase onset was delayed in both *rpt-6(RNAi)* (*p =* 3.7 × 10^−6^ compared to wild-type embryos grown at 20 °C) and c-LβL treated embryos (*p =* 5.1 × 10^−4^ compared to DMSO controls). Spindle positioning was also delayed in both *rpt-6(RNAi)* (*p =* 3.2 × 10^−6^) and c-LβL treated embryos (*p =* 0.03). Disruption of the APC delayed both anaphase onset timing (*mat-3(or180) p =* 1.3 × 10^−15^; compared to wild-type embryos quickly shifted to 25 °C), (*mat-1(RNAi) p =* 7.5 × 10^−14^ compared to wild-type embryos grown at 20 °C), and spindle position timing (*mat-3(or180) p =* 0.01; *mat-1(RNAi) p =* 5.5 × 10^−5^). RNAi targeting *fzy-1* delayed both anaphase onset (*p =* 1.6 × 10^−26^ compared to wild-type embryos grown at 20 °C) and spindle displacement (*p =* 6.2 × 10^−4^).

For experiments represented in Figure 3, the *p*-values and *n*-values were the following: After flavopiridol treatment after NEBD, anaphase onset (*p =* 3.0 × 10^−11^) and spindle displacement (*p =* 0.02) occurred earlier than in DMSO-treated wild-type. For flavopiridol treatment at the start of NEBD, anaphase onset and spindle displacement occurred earlier than wild-type (*p =* 3.8 × 10^−6^, 9.9 × 10^−3^, respectively), and also earlier than flavopiridol treatment after NEBD (*p =* 2.2 × 10^−3^, 3.5 × 10^−3^, respectively). For olomoucine II treatment after NEBD, anaphase onset (*p =* 1.0 × 10^−6^) and spindle displacement (*p =* 5.9 × 10^−3^) occurred earlier than in DMSO-treated wild-type.

For experiments represented in Figure 4, the *p*-values and *n*-values were the following: Flavopiridol treatment rescued most of the delay of anaphase onset (*p =* 1.3 × 10^−5^) and spindle displacement (*p =* 9.6 × 10^−3^) induced by the proteasome inhibitor c-LβL. The delay was not completely rescued compared to flavopiridol treatment alone (*p =* 4.2 × 10^−7^ for anaphase; *p =* 0.023 for spindle displacement). Flavopiridol treatment rescued the delay induced by *mat-1(RNAi)*, for both anaphase onset (*p =* 1.5 × 10^−7^) and spindle displacement (*p =* 1.3 × 10^−3^). The rescue timing was not statistically distinguishable from flavopiridol treatment alone (*p =* 0.078 for anaphase onset; *p =* 0.60 for spindle positioning).

For chromosome congression measurement at the time of spindle displacement in Figure 6, metaphase plates in embryos treated with flavopiridol after NEBD (*n =* 7) were not as compact as WT embryos (*n =* 21) (*p =* 7.0 × 10^−3^).

## Supporting Information

Figure S1 Details of Method for Time-Resolved Quantification of Chromatin Position and Degree of Congression At each timepoint, we extracted both the position of the chromatin and its degree of congression from an anterior-posterior linescan of fluorescence intensities through the entire width of the chromatin, from histone H2B:GFP and gamma-tubulin:GFP-expressing embryos. Fluorescence intensities are calculated as pixel intensities minus embryonic background pixel intensities, expressed in arbitrary units (a.u.).
(A) Time series through 75 s of chromosome congression, beginning 65 s after NEBD. Linescans of raw data (top), with each colored line representing one timepoint. At each timepoint, the position along the anteroposterior axis of the most concentrated mass of chromosomes was calculated by finding the peak value from a 13-pixel running average (“narrow running average”, middle graph) of the raw data. This 13-pixel width was chosen because it is roughly the smallest width of a metaphase plate. The 13-pixel running average produces a peak that we have observed to exclude chromosomes that are separated from the main mass of chromosomes just before congression is completed. Dots mark the peaks (defined by local maxima) of the running averages. For identifying anteroposterior position of the chromatin at anaphase onset, the running average window was narrowed to 5 pixels for just the timepoint when anaphase was first observed, so that the initial chromosome separation at anaphase could be resolved at the earliest possible timepoint. We used a 31-pixel running average (“wide running average,” bottom) of the raw data to identify the center of the entire chromatin mass. We then quantified the ratio of central (inner) versus neighboring (outer) average fluorescence intensity using the peak of the 31-pixel-wide running average to center the measurements.
(B) and (C) demonstrate how regions were chosen for these fluorescence intensity ratio measurements, for two individual timepoints selected from (A). (B) is the 45-s timepoint, and (C) is the 65-s timepoint. When the narrow running average has a peak and a shoulder, representing a nearly complete metaphase plate and one or more chromosomes not yet associated with it, as in (B), the peaks of the narrow and wide running averages misalign (red vertical line, marking peak of wide running average, is distant from peak of narrow running average).
These peaks align at metaphase (C). As expected, we found that this makes the ratio especially sensitive to single chromosomes that have not yet congressed to a nearly complete metaphase plate. The parameters above were developed and optimized on one set of test recordings before using them to analyze data from other recordings.
(5.45 MB EPS)

Figure S2 The APC and Cdc20/Fizzy Are Required for Timely Spindle Displacement: Frames from Films Images of embryos monitored for spindle displacement, illustrating treatments from Figure 2 that targeted the APC and Cdc20/Fizzy and delayed spindle displacement. Time is indicated in seconds after NEBD, with the same timepoints chosen as those shown in Figure 2B, and the chromatin position (green arrow) and 50% embryo length (yellow arrow) are indicated at the first of these timepoints at which spindle displacement was detectable for each treatment.
(7.06 MB EPS)

Figure S3 A Partially CDK-Independent Effect on Spindle Displacement by Misregulation of FZY-1 (A) Delay in spindle displacement in *mdf-2(av16)*, *mdf-3(av20)*, and *fzy-1(av15gf)* alleles (*mdf-2(av16) p =* 2.4 × 10^−4^; *mfd-3(av20) p =* 5.5 × 10^−6^; *fzy-1(av15gf) p =* 9.2 × 10^−5^), compared to wild-type embryos that were grown at 24 °C.
(B) Flavopiridol partially rescued these delays (*mdf-2(av16) p =* 0.014; *mfd-3(av20) p =* 2.9 × 10^−3^; *fzy-1(av15gf) p =* 4.9 × 10^−3^), and shortened the time to anaphase onset (*mdf-2(av16) p =* 2.9 × 10^−5^; *mfd-3(av20) p =* 1.9 × 10^−8^; *fzy-1(av15gf) p =* 1.2 × 10^−9^). The time to anaphase onset for each mutant is not statistically distinguishable from flavopiridol treatment alone (*mdf-2(av16) p =* 0.056; *mfd-3(av20) p =* 0.91; *fzy-1(av15gf) p =* 0.73). Spindle displacement timing was incompletely rescued in two of these flavopiridol-treated mutants, compared to drug treatment alone (*mdf-2(av16) p =* 0.14; *mfd-3(av20) p =* 0.011; *fzy-1(av15gf) p =* 1.1 × 10^−3^). Number of cases analyzed for each experiment is indicated in parentheses on the anaphase onset bars. Error bars represent the 95% confidence intervals for significance, and asterisks represent statistical significance at *p* < 0.05.
(68.12 KB EPS)

Video S1 The Mitotic Spindle Begins to Shift Approximately 10 s after the Completion of Chromosome Congression Movie of the embryo from Figure 1B–1D expressing histone H2B:GFP and gamma-tubulin:GFP, and a dynamically annotated version of the corresponding graph. Dotted line in the movie marks 50% embryo length. As the fluorescence ratio in the center of the spindle to just outside this region peaks at metaphase, displacement of the spindle with a compact metaphase plate is evident in the embryo and the graph. Light blue datapoints in this graph indicate the center of the two chromatin masses after anaphase.
(1.97 MB MOV)

Video S2 Disruption of Proteasome Activity Delayed Both Spindle Displacement and Anaphase Onset Three cases of each treatment: wild-type untreated (top three), *rpt-6(RNAi)* (middle three), and c-LβL (bottom three). Small regions of each embryo are shown, each through most of the mitotic spindle. The red line is 50% embryo length, and each movie starts at NEBD.
(3.53 MB MOV)

Video S3 Premature CDK Inactivation Caused Premature Spindle Displacement and Anaphase Onset Three cases of each, untreated (top three) and flavopiridol-treated (bottom three), are shown as in Video S2. Blank frames are shown at time of flavopiridol-treatment.
(911 KB MOV)

Video S4 Anaphase Timing Is Unaffected When Spindle Displacement Is Prevented WT is wild-type control. Spindle displacement was prevented by *par-2* RNAi or *gpr-1/2* RNAi.
(1.36 MB MOV)

Video S5 Flavopiridol Treatment as NEBD Began Resulted in Chromosomes Being Spread over a Significantly Wider Area Than Normal at the Time of Spindle Displacement An embryo laser-permeabilized to flavopiridol as NEBD began (right). Spindle displacement toward the posterior (top) and anaphase both occur before chromosome congression is complete in the flavopiridol-treated embryo. The embryo in the right is the bottom embryo among the flavopiridol-treated embryos in Figure 6A. The embryo on the left was laser-permeabilized to flavopiridol later than the embryo on the right was, and chromosome dynamics appear less affected as expected.
(4.55 MB MOV)

Video S6 Click here for additional data file. Flavopiridol Treatment Directly after the Start of NEBD Resulted Sometimes in Individual Chromosomes Failing to Associate with a Metaphase Plate by the Time of Premature Spindle Displacement
As in Video S5, but this is a treated embryo alone, with an apparently less severe defect. One chromosome is not associated with the metaphase plate during spindle displacement and anaphase. This embryo is also shown as the center embryo among the flavopiridol-treated embryos in Figure 6A.
(4.08 MB MOV)

## Abbreviations

APC: anaphase-promoting complex
CDK: cyclin-dependent kinase
GFP: green fluorescent protein
NEBD: nuclear envelope breakdown
c-LβL: *clasto*-lactacystin β-lactone

## References

[pbio-1000088-b001] Whittaker JR (1980). Acetylcholinesterase development in extra cells caused by changing the distribution of myoplasm in ascidian embryos. J Embryol Exp Morphol.

[pbio-1000088-b002] Watt FM, Hogan BL (2000). Out of Eden: stem cells and their niches. Science.

[pbio-1000088-b003] Brunet S, Maro B (2005). Cytoskeleton and cell cycle control during meiotic maturation of the mouse oocyte: integrating time and space. Reproduction.

[pbio-1000088-b004] Lillie F (1901). The organization of the egg of Unio, based on a study of its maturation, fertilization, and cleavage. J Morphol.

[pbio-1000088-b005] Wilson EB (1896). The cell in development and inheritance.

[pbio-1000088-b006] Gonczy P (2008). Mechanisms of asymmetric cell division: flies and worms pave the way. Nat Rev Mol Cell Biol.

[pbio-1000088-b007] Grill SW, Gonczy P, Stelzer EH, Hyman AA (2001). Polarity controls forces governing asymmetric spindle positioning in the Caenorhabditis elegans embryo. Nature.

[pbio-1000088-b008] Grill SW, Howard J, Schaffer E, Stelzer EH, Hyman AA (2003). The distribution of active force generators controls mitotic spindle position. Science.

[pbio-1000088-b009] Labbe JC, McCarthy EK, Goldstein B (2004). The forces that position a mitotic spindle asymmetrically are tethered until after the time of spindle assembly. J Cell Biol.

[pbio-1000088-b010] Labbe JC, Maddox PS, Salmon ED, Goldstein B (2003). PAR proteins regulate microtubule dynamics at the cell cortex in C. elegans. Curr Biol.

[pbio-1000088-b011] Kozlowski C, Srayko M, Nedelec F (2007). Cortical microtubule contacts position the spindle in C. elegans embryos. Cell.

[pbio-1000088-b012] Tsou MF, Hayashi A, Rose LS (2003). LET-99 opposes Galpha/GPR signaling to generate asymmetry for spindle positioning in response to PAR and MES-1/SRC-1 signaling. Development.

[pbio-1000088-b013] Furuta T, Tuck S, Kirchner J, Koch B, Auty R (2000). EMB-30: an APC4 homologue required for metaphase-to-anaphase transitions during meiosis and mitosis in Caenorhabditis elegans. Mol Biol Cell.

[pbio-1000088-b014] Golden A, Sadler PL, Wallenfang MR, Schumacher JM, Hamill DR (2000). Metaphase to anaphase (mat) transition-defective mutants in Caenorhabditis elegans. J Cell Biol.

[pbio-1000088-b015] Kitagawa R, Law E, Tang L, Rose AM (2002). The Cdc20 homolog, FZY-1, and its interacting protein, IFY-1, are required for proper chromosome segregation in Caenorhabditis elegans. Curr Biol.

[pbio-1000088-b016] Cowan CR, Hyman AA (2004). Asymmetric cell division in C. elegans: cortical polarity and spindle positioning. Annu Rev Cell Dev Biol.

[pbio-1000088-b017] Oegema K, Hyman AA (2006). Cell division. WormBook.

[pbio-1000088-b018] Lyczak R, Gomes JE, Bowerman B (2002). Heads or tails: cell polarity and axis formation in the early Caenorhabditis elegans embryo. Dev Cell.

[pbio-1000088-b019] Munro E (2007). The microtubules dance and the spindle poles swing. Cell.

[pbio-1000088-b020] Nguyen-Ngoc T, Afshar K, Gonczy P (2007). Coupling of cortical dynein and G alpha proteins mediates spindle positioning in Caenorhabditis elegans. Nat Cell Biol.

[pbio-1000088-b021] Oegema K, Desai A, Rybina S, Kirkham M, Hyman AA (2001). Functional analysis of kinetochore assembly in Caenorhabditis elegans. J Cell Biol.

[pbio-1000088-b022] Gutierrez GJ, Ronai Z (2006). Ubiquitin and SUMO systems in the regulation of mitotic checkpoints. Trends Biochem Sci.

[pbio-1000088-b023] Bowerman B, Kurz T (2006). Degrade to create: developmental requirements for ubiquitin-mediated proteolysis during early C. elegans embryogenesis. Development.

[pbio-1000088-b024] Dick LR, Cruikshank AA, Destree AT, Grenier L, McCormack TA (1997). Mechanistic studies on the inactivation of the proteasome by lactacystin in cultured cells. J Biol Chem.

[pbio-1000088-b025] Gonczy P, Echeverri C, Oegema K, Coulson A, Jones SJ (2000). Functional genomic analysis of cell division in C. elegans using RNAi of genes on chromosome III. Nature.

[pbio-1000088-b026] Pines J (2006). Mitosis: a matter of getting rid of the right protein at the right time. Trends Cell Biol.

[pbio-1000088-b027] Liu J, Vasudevan S, Kipreos ET (2004). CUL-2 and ZYG-11 promote meiotic anaphase II and the proper placement of the anterior-posterior axis in C. elegans. Development.

[pbio-1000088-b028] Sonneville R, Gonczy P (2004). Zyg-11 and cul-2 regulate progression through meiosis II and polarity establishment in C. elegans. Development.

[pbio-1000088-b029] Boxem M, Srinivasan DG, van den Heuvel S (1999). The Caenorhabditis elegans gene ncc-1 encodes a cdc2-related kinase required for M phase in meiotic and mitotic cell divisions, but not for S phase. Development.

[pbio-1000088-b030] Potapova TA, Daum JR, Pittman BD, Hudson JR, Jones TN (2006). The reversibility of mitotic exit in vertebrate cells. Nature.

[pbio-1000088-b031] Sedlacek HH (2001). Mechanisms of action of flavopiridol. Crit Rev Oncol Hematol.

[pbio-1000088-b032] Munoz MJ, Santori MI, Rojas F, Gomez EB, Tellez-Inon MT (2006). Trypanosoma cruzi Tcp12CKS1 interacts with parasite CRKs and rescues the p13SUC1 fission yeast mutant. Mol Biochem Parasitol.

[pbio-1000088-b033] Stemmann O, Gorr IH, Boos D (2006). Anaphase topsy-turvy: Cdk1 a securin, separase a CKI. Cell Cycle.

[pbio-1000088-b034] Vermeulen K, Van Bockstaele DR, Berneman ZN (2003). The cell cycle: a review of regulation, deregulation and therapeutic targets in cancer. Cell Prolif.

[pbio-1000088-b035] May KM, Hardwick KG (2006). The spindle checkpoint. J Cell Sci.

[pbio-1000088-b036] Musacchio A, Salmon ED (2007). The spindle-assembly checkpoint in space and time. Nat Rev Mol Cell Biol.

[pbio-1000088-b037] Li R, Murray AW (1991). Feedback control of mitosis in budding yeast. Cell.

[pbio-1000088-b038] Hoyt MA, Totis L, Roberts BT (1991). S. cerevisiae genes required for cell cycle arrest in response to loss of microtubule function. Cell.

[pbio-1000088-b039] Hardwick KG, Johnston RC, Smith DL, Murray AW (2000). MAD3 encodes a novel component of the spindle checkpoint which interacts with Bub3p, Cdc20p, and Mad2p. J Cell Biol.

[pbio-1000088-b040] Encalada SE, Willis J, Lyczak R, Bowerman B (2005). A spindle checkpoint functions during mitosis in the early Caenorhabditis elegans embryo. Mol Biol Cell.

[pbio-1000088-b041] Stein KK, Davis ES, Hays T, Golden A (2007). Components of the spindle assembly checkpoint regulate the anaphase-promoting complex during meiosis in Caenorhabditis elegans. Genetics.

[pbio-1000088-b042] Clarke DJ, Segal M, Andrews CA, Rudyak SG, Jensen S (2003). S-phase checkpoint controls mitosis via an APC-independent Cdc20p function. Nat Cell Biol.

[pbio-1000088-b043] Lew DJ, Burke DJ (2003). The spindle assembly and spindle position checkpoints. Annu Rev Genet.

[pbio-1000088-b044] O'Connell CB, Wang YL (2000). Mammalian spindle orientation and position respond to changes in cell shape in a dynein-dependent fashion. Mol Biol Cell.

[pbio-1000088-b045] Goldstein B, Macara IG (2007). The PAR proteins: fundamental players in animal cell polarization. Dev Cell.

[pbio-1000088-b046] Bowman SK, Neumuller RA, Novatchkova M, Du Q, Knoblich JA (2006). The Drosophila NuMA Homolog Mud regulates spindle orientation in asymmetric cell division. Dev Cell.

[pbio-1000088-b047] Colombo K, Grill SW, Kimple RJ, Willard FS, Siderovski DP (2003). Translation of polarity cues into asymmetric spindle positioning in Caenorhabditis elegans embryos. Science.

[pbio-1000088-b048] Du Q, Macara IG (2004). Mammalian Pins is a conformational switch that links NuMA to heterotrimeric G proteins. Cell.

[pbio-1000088-b049] Gotta M, Dong Y, Peterson YK, Lanier SM, Ahringer J (2003). Asymmetrically distributed C. elegans homologs of AGS3/PINS control spindle position in the early embryo. Curr Biol.

[pbio-1000088-b050] Izumi Y, Ohta N, Hisata K, Raabe T, Matsuzaki F (2006). Drosophila Pins-binding protein Mud regulates spindle-polarity coupling and centrosome organization. Nat Cell Biol.

[pbio-1000088-b051] Park DH, Rose LS (2008). Dynamic localization of LIN-5 and GPR-1/2 to cortical force generation domains during spindle positioning. Dev Biol.

[pbio-1000088-b052] Siller KH, Cabernard C, Doe CQ (2006). The NuMA-related Mud protein binds Pins and regulates spindle orientation in Drosophila neuroblasts. Nat Cell Biol.

[pbio-1000088-b053] Srinivasan DG, Fisk RM, Xu H, van den Heuvel S (2003). A complex of LIN-5 and GPR proteins regulates G protein signaling and spindle function in C elegans. Genes Dev.

[pbio-1000088-b054] Couwenbergs C, Labbe JC, Goulding M, Marty T, Bowerman B (2007). Heterotrimeric G protein signaling functions with dynein to promote spindle positioning in C. elegans. J Cell Biol.

[pbio-1000088-b055] Tsou MF, Hayashi A, DeBella LR, McGrath G, Rose LS (2002). LET-99 determines spindle position and is asymmetrically enriched in response to PAR polarity cues in C. elegans embryos. Development.

[pbio-1000088-b056] Gehmlich K, Haren L, Merdes A (2004). Cyclin B degradation leads to NuMA release from dynein/dynactin and from spindle poles. EMBO Rep.

[pbio-1000088-b057] Mishima M, Pavicic V, Gruneberg U, Nigg EA, Glotzer M (2004). Cell cycle regulation of central spindle assembly. Nature.

[pbio-1000088-b058] Sullivan M, Lehane C, Uhlmann F (2001). Orchestrating anaphase and mitotic exit: separase cleavage and localization of Slk19. Nat Cell Biol.

[pbio-1000088-b059] Tsou MF, Stearns T (2006). Mechanism limiting centrosome duplication to once per cell cycle. Nature.

[pbio-1000088-b060] Bembenek JN, Richie CT, Squirrell JM, Campbell JM, Eliceiri KW (2007). Cortical granule exocytosis in C. elegans is regulated by cell cycle components including separase. Development.

[pbio-1000088-b061] Grava S, Schaerer F, Faty M, Philippsen P, Barral Y (2006). Asymmetric recruitment of dynein to spindle poles and microtubules promotes proper spindle orientation in yeast. Dev Cell.

[pbio-1000088-b062] Liakopoulos D, Kusch J, Grava S, Vogel J, Barral Y (2003). Asymmetric loading of Kar9 onto spindle poles and microtubules ensures proper spindle alignment. Cell.

[pbio-1000088-b063] Maekawa H, Usui T, Knop M, Schiebel E (2003). Yeast Cdk1 translocates to the plus end of cytoplasmic microtubules to regulate bud cortex interactions. EMBO J.

[pbio-1000088-b064] Gonczy P, Pichler S, Kirkham M, Hyman AA (1999). Cytoplasmic dynein is required for distinct aspects of MTOC positioning, including centrosome separation, in the one cell stage Caenorhabditis elegans embryo. J Cell Biol.

[pbio-1000088-b065] Hartwell LH, Weinert TA (1989). Checkpoints: controls that ensure the order of cell cycle events. Science.

[pbio-1000088-b066] Holway AH, Kim SH, La Volpe A, Michael WM (2006). Checkpoint silencing during the DNA damage response in Caenorhabditis elegans embryos. J Cell Biol.

[pbio-1000088-b067] Brauchle M, Baumer K, Gonczy P (2003). Differential activation of the DNA replication checkpoint contributes to asynchrony of cell division in C. elegans embryos. Curr Biol.

[pbio-1000088-b068] Goto M, Eddy EM (2004). Speriolin is a novel spermatogenic cell-specific centrosomal protein associated with the seventh WD motif of Cdc20. J Biol Chem.

[pbio-1000088-b069] Reimann JD, Gardner BE, Margottin-Goguet F, Jackson PK (2001). Emi1 regulates the anaphase-promoting complex by a different mechanism than Mad2 proteins. Genes Dev.

[pbio-1000088-b070] Song MS, Song SJ, Ayad NG, Chang JS, Lee JH (2004). The tumour suppressor RASSF1A regulates mitosis by inhibiting the APC-Cdc20 complex. Nat Cell Biol.

[pbio-1000088-b071] Wang Q, Moyret-Lalle C, Couzon F, Surbiguet-Clippe C, Saurin JC (2003). Alterations of anaphase-promoting complex genes in human colon cancer cells. Oncogene.

[pbio-1000088-b072] Kaltschmidt JA, Davidson CM, Brown NH, Brand AH (2000). Rotation and asymmetry of the mitotic spindle direct asymmetric cell division in the developing central nervous system. Nat Cell Biol.

[pbio-1000088-b073] Zhang SO, Weisblat DA (2005). Applications of mRNA injections for analyzing cell lineage and asymmetric cell divisions during segmentation in the leech Helobdella robusta. Development.

[pbio-1000088-b074] Roegiers F, Jan YN (2004). Asymmetric cell division. Curr Opin Cell Biol.

[pbio-1000088-b075] Ren X, Weisblat DA (2006). Asymmetrization of first cleavage by transient disassembly of one spindle pole aster in the leech Helobdella robusta. Dev Biol.

[pbio-1000088-b076] Ishii R, Shimizu T (1995). Unequal first cleavage in the *Tubifex* egg: involvement of a monastral mitotic apparatus. Dev Growth Differ.

[pbio-1000088-b077] Shimizu T (1996). The first two cleavages in *Tubifex* involve distinct mechanisms to generate asymmetry in mitotic apparatus. Hydrobiologia.

[pbio-1000088-b078] Verlhac MH, Lefebvre C, Guillaud P, Rassinier P, Maro B (2000). Asymmetric division in mouse oocytes: with or without Mos. Curr Biol.

[pbio-1000088-b079] Fire A, Xu S, Montgomery MK, Kostas SA, Driver SE (1998). Potent and specific genetic interference by double-stranded RNA in Caenorhabditis elegans. Nature.

[pbio-1000088-b080] Kamath RS, Martinez-Campos M, Zipperlen P, Fraser AG, Ahringer J (2001). Effectiveness of specific RNA-mediated interference through ingested double-stranded RNA in Caenorhabditis elegans. Genome Biol.

